# Quality of Life in Heart Failure: An Important Goal in Treatment

**DOI:** 10.36660/abc.20190741

**Published:** 2020-01

**Authors:** Brenno Rizerio Gomes, Edimar Alcides Bocchi

**Affiliations:** 1 Unidade de Insuficiência Cardíaca e Dispositivos de Assistência Circulatória Mecânica do Instituto do Coração do Hospital das Clínicas da Faculdade de Medicina da Universidade de São Paulo (InCor HCFMUSP), São Paulo, SP - Brazil; 2 Departamento de Cardiopneumologia da Faculdade de Medicina da Universidade de São Paulo, São Paulo, SP - Brazil

**Keywords:** Heart Failure, Anxiety/diagnosis, Hospitalization, Quality of Life, Aged, Stroke Volume

Brazil is the country with the highest prevalence of anxiety disorders, according to the World Health Organization and ranks 5^th^ regarding the prevalence of depression.^1^ Mood disorders, which include anxiety and depression, are often neglected in clinical practice,^2,3^ and their diagnosis in patients with heart failure (HF) is even more challenging, given the overlap of several symptoms, such as fatigue, weight loss and sleep disorders.^4,5^

In this issue of the Archives, the cross-sectional study by Figueiredo et al.^6^ evaluated, in a population of 99 patients with HF and reduced ejection fraction, which clinical, sociodemographic and psychological variables most correlated with the quality of life assessed by the Minnesota Living with Heart Failure Questionnaire. The main factors associated with poorer quality of life were dyspnea advanced functional class (New York Heart Association III and IV), previous hospitalization and anxiety symptoms. Depression was not independently associated with reduced quality of life, but several other studies have found this association.^7,8^ The study also shows an alarming prevalence of anxiety symptoms in these patients, of 50%, when compared to 9.3% in the overall population.^1^

The interaction between cardiovascular disease and mood disorders occurs in a bi-direction manner.^9^ Recently, it was described that optimism is associated with lower risk of cardiovascular events and mortality from any cause.^10^ The risk of developing HF in patients with depression is 1.5 to 2.6-fold higher than in the overall population.^11^ In individuals diagnosed with HF, depression indicates a worse prognosis and is associated with higher hospitalization and mortality rates.^11^ Possible mechanisms to explain this association involve lower adherence to pharmacological and non-pharmacological treatment in patients with depression and greater tendency towards having unhealthy lifestyles.^12,13^ The more advanced the dyspnea functional class, the worse the symptoms of depression and the quality of life.^8,11^

Regarding anxiety disorders, affected individuals also seem to have a higher risk of developing HF throughout life.^14^ In those diagnosed with HF, the presence of anxiety is associated with poorer quality of life;^15^ however, the correlation with increased mortality is not as well established.^16,17^

Evidence is limited for the treatment of mood disorders in HF patients. Cognitive behavioral therapy was tested in a randomized study of 158 patients diagnosed with major depression and heart failure.18 Psychotherapy was associated with remission of depression (46% vs. 19%, NNT = 3.8), in addition to improvement in quality of life, anxiety and fatigue.

The pharmacological treatment of choice for mood disorders consists in selective serotonin reuptake inhibitors.^19,20^ For patients with HF and reduced ejection fraction, two prominent randomized trials tested these therapies in individuals with major depression: 1) MOOD-HF,^21^ which included 372 patients to receive escitalopram or placebo for 3 months, and 2) SADHART-CHF,^22^ which included 469 patients to receive sertraline or placebo for 18 months. Both were negative for the primary outcome, showing no benefit of pharmacological therapies in the treatment of depression in HF patients.

A structured and multidisciplinary HF management education and care program implemented in 350 patients in our service has shown a reduction in unplanned hospitalizations^23^ and improved quality of life, especially in the emotional domain,^23,24^ suggesting that this approach may be beneficial for patients with mood disorders.

The study by Figueiredo et al.,^6^ suffers from the usual limitations of a single-center, cross-sectional and observational assessment, and the small number of patients prevents more robust conclusions. The assessed primary outcome was quality of life, but it remains to be prospectively seen whether anxiety has an impact on clinical outcomes, such as hospital admissions or mortality.

In conclusion, the present article by Figueiredo et al.^6^ reinforces the importance of a holistic approach for HF patients, by demonstrating that neglected factors such as anxiety disorders are very prevalent in this population and may have an impact on quality of life. The field of treatment for mood disorders has been little explored and deserves further attention in future randomized trials.
